# Artificial multiferroic heterostructures—electric field effects and their perspectives

**DOI:** 10.1080/14686996.2024.2412970

**Published:** 2024-11-05

**Authors:** Tomoyasu Taniyama, Yoshihiro Gohda, Kohei Hamaya, Takashi Kimura

**Affiliations:** a Department of Physics, Nagoya University, Nagoya, Japan; b Department of Materials Science and Engineering, Tokyo Institute of Technology, Yokohama, Japan; c Center for Spintronics Research Network, Graduate School of Engineering Science, Osaka University, Toyonaka, Japan; d Department of Physics, Kyushu University, Fukuoka, Japan

**Keywords:** Multiferroics, ferromagnetic/ferroelectric heterostructures, magneto-electric coupling, spintronics

## Abstract

Artificial multiferroic heterostructures, that is to say, ferromagnetic/ferroelectric heterostructures, have been the subject of considerable research interest as a potential material basis for the creation of novel energy-efficient device applications. Given that polarization reversal occurs in ferroelectric materials when an electric field is applied, it is possible to modulate the magnetic properties of a ferromagnetic layer due to changes in the polarization charge associated with a ferroelectric material, or due to exchange coupling, ionic transport, or orbital hybridization at the interface between the ferromagnetic and ferroelectric materials. Another essential characteristic of ferroelectric materials is their inverse piezoelectricity, which induces strain through the application of an electric field. The inverse piezoelectric strain is transferred to the ferromagnetic layer, thereby modulating the magnetic properties due to the magnetoelastic effect. In comparison to the various effects, the influence of strain transfer on magnetic properties is particularly pronounced, offering promising avenues for controlling magnetic properties via an electric field without the use of an electric current. This review article aims to present an overview of recent developments in the field of electric field effects on magnetic properties, with a particular focus on the role of strain transfer in magneto-electric effects. The potential applications of artificial multiferroic heterostructures are discussed, including the control of magnetic anisotropy, as well as the manipulation of perpendicular magnetic anisotropy, magnetoresistance, interlayer exchange coupling, spin wave propagation, spin damping, magnetic phase, and superconductivity. The article concludes with a consideration of the future prospects of artificial multiferroic heterostructures for next-generation device applications.

## IMPACT STATEMENT

This article provides a comprehensive review of the recent progress in the fabrication of high-performance artificial multiferroic heterostructures and their potential for next-generation energy-efficient device applications.

## GRAPHICAL ABSTRACT

**Figure:**
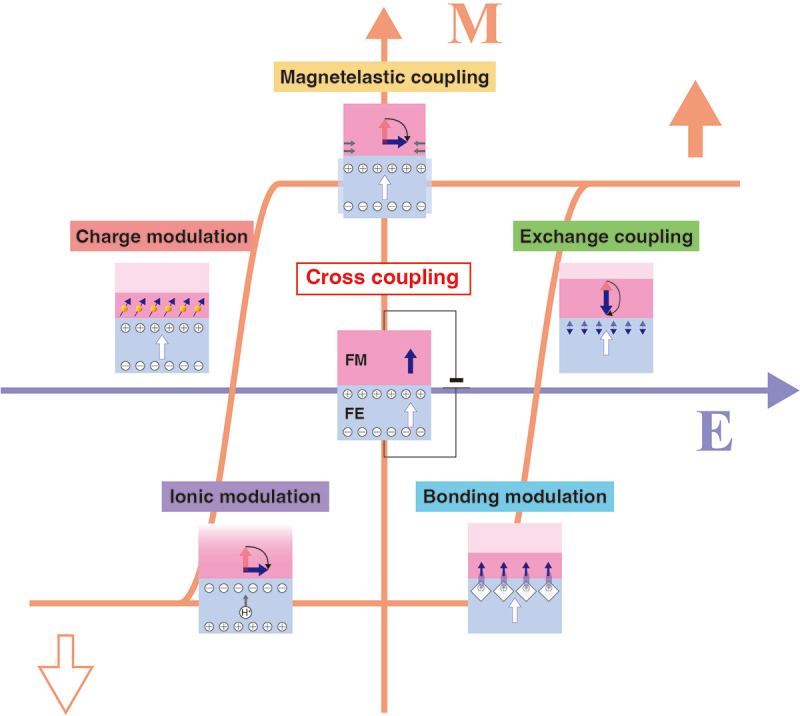

## 1. Introduction

In recent years, there has been a growing demand for energy-efficient technology that controls the magnetic and transport properties of magnetic materials, with the aim of developing low-power spintronic and electronic devices. The switching of the magnetization orientation of a magnetic layer in a spintronic memory device has already been demonstrated using an electric current-based approach, for example, spin transfer torque and spin orbit torque [1–5]. This phenomenon occurs when the transfer of spin angular momentum from conduction electrons to local magnetic moments exerts a torque on the net magnetization. Nevertheless, the utilization of an electric current inevitably results in a considerable consumption of electrical power due to Joule heating, which presents a significant challenge for the development of new technologies that enable the control of magnetic properties without an electric current.

**Figure 1.**
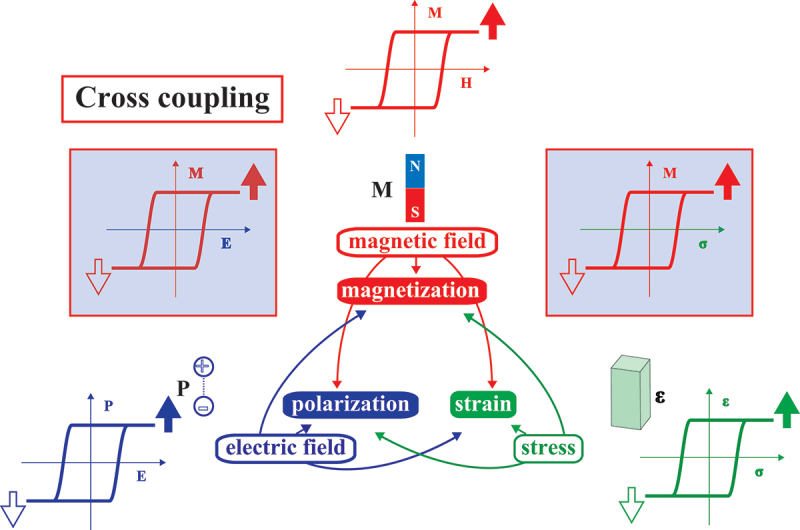
Cross-coupling in multiferroic materials. Modified after [6]. Copyright 2015 IOP Publishing.

An encouraging approach is the use of multiferroic materials, which exhibit both magnetic and electric orders within a single material, as illustrated in Figure 1 [7–11]. It has been demonstrated that there is a direct cross-coupling between magnetic order and electric order in type II multiferroics via antisymmetric exchange interaction [6], which leads to the generation of electric polarization due to the spiral spin configuration of the nearest spins [12]. One of the most significant advantages of multiferroics is that they do not necessitate the flow of an electric current to control their magnetic properties. This stands in stark contrast to the current-based approach, such as spin transfer torque. Instead, an electric field is applied to manipulate the magnetic characteristics of multiferroics, thereby markedly reducing power consumption. As a consequence of this remarkable advantage, a variety of single-phase multiferroics have been investigated since the discovery of RMnO${\;}_{3}$ (R: rare earth element), including TbMnO${\;}_{3}$ [7] and DyMnO${\;}_{3}$ [13]. Despite the intriguing magneto-electric (ME) coupling, the magnetic order in a single-phase multiferroic material is typically antiferromagnetic (AFM), and the magnetic-phase transition temperature is also considerably below room temperature, which renders room temperature device applications challenging. For further details on single-phase multiferroics, including split-order type-I multiferroics, refer to the comprehensive review articles [14–18].

**Figure 2.**
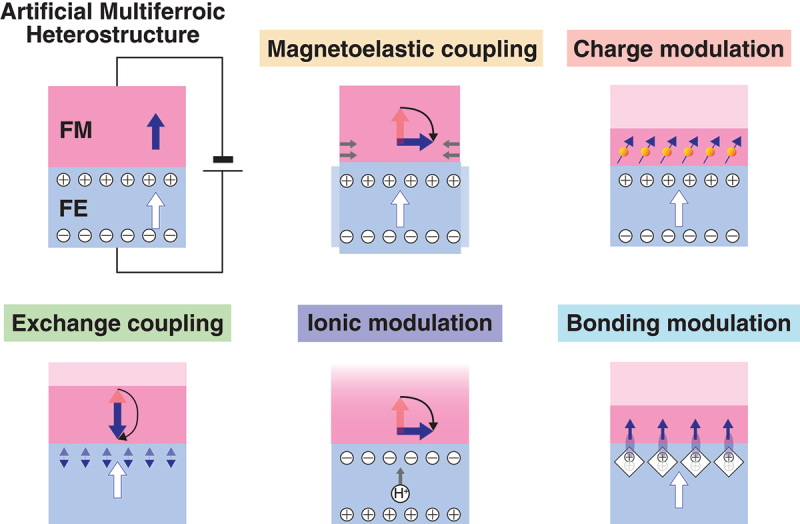
Artificial multiferroic heetrostructures and the mechanisms underlying ME coupling effects.

Another promising material is an artificial multiferroic heterostructure, which is a two-phase system composed of a ferromagnetic (FM) material and a ferroelectric (FE) material as depicted in Figure 2. In light of the fact that the spatial inversion symmetry is broken at the FM/FE interface, it can be anticipated that multiferroic properties will emerge in the FM region with broken time reversal symmetry in the vicinity of the interface. Any combination of FM and FE materials may be used to construct the artificial multiferroic heterostructure, thus facilitating the creation of room-temperature FM and FE multiferroic materials. To date, a substantial number of material combinations have been investigated for the potential application of artificial multiferroic heterostructures, as detailed in Table 1.

**Table 1.** Magneto-electric coupling effect in various artificial multiferroic heterostructures.

Mechanism	Quantity	Ferroelectric or ionic material	Ferromagnetic material	ME coefficient*	Ref.
Charge	Magnetic order	BaTiO${\;}_{3}$	SrRuO${\;}_{3}$	–	[19]
Charge	Magnetic order	PZT	La${\;}_{1-x}$Sr${\;}_{x}$MnO${\;}_{3}$	1.35	[20]
Magnetoelastic	Magnetic order	BaTiO${\;}_{3}$	FeRh	1600	[21]
Magnetoelastic	Magnetic anisotropy	BaTiO${\;}_{3}$	Ni	0.05	[22]
Magnetoelastic	Magnetic anisotropy	BaTiO${\;}_{3}$	La${\;}_{1-x}$Sr${\;}_{x}$MnO${\;}_{3}$	2-23	[23]
Magnetoelastic	Magnetic anisotropy	PZT	Ni	–	[24]
Magnetoelastic	Magnetic anisotropy	PMN-PT	CoFeB	800	[25]
Magnetoelastic	Magnetic anisotropy	PMN-PT	Co${\;}_{2}$FeSi	1000	[26]
Magnetoelastic	Magnetic anisotropy	PMN-PT	Fe${\;}_{1-x}$Ga${\;}_{x}$	2000	[27]
Magnetoelastic	Perpendicular magnetic anisotropy	BaTiO${\;}_{3}$	[Cu/Ni]${\;}_{n}$	60	[28]
Magnetoelastic	Perpendicular magnetic anisotropy	PMN-PT	[Co/Pd]${\;}_{n}$	110	[29]
Magnetoelastic	Perpendicular magnetic anisotropy	PMN-PT	[Co/Pt]${\;}_{n}$	–	[30]
Magnetoelastic	Perpendicular magnetic anisotropy	PMN-PT	FePt	–	[31]
Magnetoelastic	Domain wall motion	BaTiO${\;}_{3}$	Fe	–	[32]
Magnetoelastic	Domain wall motion	BaTiO${\;}_{3}$	Fe${\;}_{3}$O${\;}_{4}$	–	[33]
Magnetoelastic	Domain wall motion	PZT	IrMn/Co/Cu/CoFeB/MgO	–	[34]
Magnetoelastic	Spin wave	BaTiO${\;}_{3}$	Fe	–	[35]
Magnetoelastic	Interlayer exchange	PMN-PT	Co/Ru/Co	–	[36]
Magnetoelastic	Interlayer exchange	PMN-PT	FeCoB/Ru/FeCoB	–	[37]
Magnetoelastic	Skyrmion	PMN-PT	[Pt/Co/Ta]${\;}_{n}$	–	[38]
Magnetoelastic	Ferromagnetic resonance	ZN-PT	FeGaB	–	[39]
Magnetoelastic	Ferromagnetic resonance	PZN-PT	Fe${\;}_{3}$O${\;}_{4}$	–	[40]
Exchage	Magnetic anisotropy	YMnO${\;}_{3}$	NiFe	13	[41]
Exchage	Magnetic anisotropy	BiFeO${\;}_{3}$	La${\;}_{1-x}$Sr${\;}_{x}$MnO${\;}_{3}$	–	[42]
Exchage	Magnetic anisotropy	BiFeO${\;}_{3}$	NiFe	–	[43]
Exchage	Magnetic anisotropy	BiFeO${\;}_{3}$	CoFe/Cu/CoFe	10-30	[44]
Ionic	Magnetization	BaTiO${\;}_{3}$	La${\;}_{1-x}$Sr${\;}_{x}$MnO${\;}_{3}$	–	[45]
Ionic	Magnetic anisotropy	GdO${\;}_{x}$	Co	1.2	[46]
Ionic	Magnetic anisotropy	DEME-TFSI	La${\;}_{1-x}$Sr${\;}_{x}$MnO${\;}_{3}$/BaTiO${\;}_{3}$	–	[47]
Orbital hybridization	Magnetization	BaTiO${\;}_{3}$	Fe	–	[48]
Orbital hybridization	Magnetization	BaTiO${\;}_{3}$	Fe${\;}_{3}$Si	–	[49]
Orbital hybridization	Spin polarization	BaTiO${\;}_{3}$	Fe/BaTiO${\;}_{3}$/La${\;}_{1-x}$Sr${\;}_{x}$MnO${\;}_{3}$	–	[50]
Orbital hybridization	Spin polarization	PZT	Co/PZT/La${\;}_{1-x}$Sr${\;}_{x}$MnO${\;}_{3}$	–	[51]
Orbital hybridization	Topological Hall effect	BaTiO${\;}_{3}$	SrRuO${\;}_{3}$	–	[52]

*The unit of ME coefficient: 10${\;}^{-8}$s/m.

PZT: PbZr${\;}_{1-x}$Ti${\;}_{x}$O${\;}_{3}$, PMN-PT: Pb(Mg${\;}_{1/3}$Nb${\;}_{2/3}$)O${\;}_{3}$-PbTiO${\;}_{3}$, PZN-PT: Pb(Zn${\;}_{1/3}$Nb${\;}_{2/3}$)O${\;}_{3}$-PbTiO${\;}_{3}$, DEME-TFSI: N,N-diethyl-N-(2-methoxyethyl)-N-ethylammonium bis-(trifluoromethylsulfonyl)-imide.

This review presents recent developments in the fabrication of new artificial multiferroic heterostructures and their applications. We begin by summarizing the required performance of artificial multiferroic heterostructures, followed by a brief overview of the mechanisms of ME coupling in artificial multiferroic heterostructures, along with an examination of the characteristics associated with each mechanism. Subsequently, we will present a selection of illustrative examples of artificial multiferroic heterostructures that exhibit a significant ME coupling effect. We will then discuss the applications of artificial multiferroic heterostructures and conclude with a summary and outlook. Readers seeking more comprehensive and detailed information are referred to the existing literature [18,52–68].

## 2. Performance requirements for ME materials

Reducing the power consumption of electronic devices is a pressing challenge for the electronics industry. In particular, in spintronics, which is expected to be an innovative technology contributing to low power consumption, there is an urgent need to develop an energy-efficient control technology for the magnetic and transport properties that underlie all device functions. As mentioned in the previous section, electric current-based methods such as spin-transfer torque have been used in the development of ultra-low power spintronic devices, but the current density required for magnetization reversal is large at about 10${\;}^{11}$ A/m${\;}^{2}$ [69], and the development of voltage control technology for magnetic anisotropy is required to achieve even lower power consumption: voltage-controlled magnetic anisotropy (VCMA) has been extensively investigated [70,71]. However, there are many challenges to device implementation, such as the huge electric fields of MV/cm or more required in conventional ferromagnetic ultrathin film/dielectric oxide heterostructures. In contrast, artificial multiferroic heterostructures have the great advantage of being able to control the magnetization orientation at a low electric field of about kV/cm, which is more than two orders of magnitude lower than VCMA, and are very encouraging as alternative materials to achieve a breakthrough in energy-efficient control of magnetization orientation. The usefulness of artificial multiferroic heterostructures is not limited to magnetization orientation, but also includes innovative devices exploiting the electric field control functions of magnetoresistance [72,73], spin-wave transmission [35], magnetic phases [45], superconductivity [74], and even heat transport at the ferromagnetic/ferroelectric interface, as well as devices using ambient microwave energy, which is exploding with the development of wireless information and communication technology. The excitation of magnons in the ferromagnetic materials of artificial multiferroic heterostructures by harvesting ambient microwave energy is expected to lead to the development of applications such as power generation via interfacial magnon-phonon conversion. Thus, artificial multiferroic heterostructures are promising materials with a wide range of innovative applications. On the other hand, due to the high degree of freedom in material selection, no clear guideline has yet been established as to which ferromagnetic and ferroelectric materials can be combined to achieve a large magneto-electric coefficient above 10${\;}^{-5}$ s/m. Also, the almost artificial multiferroic heterostructures reported so far are composed of bulk ferroelectric substrates, presumably due to imperfections in ferroelectric thin films, so that a voltage of several tens of volts is required to switch the magnetization orientation, although the required electric field itself is in the kV/cm range. Therefore, in order to integrate artificial multiferroic heterostructures into practical device applications, a comprehensive experimental and theoretical understanding of how to design high-quality artificial multiferroic heterostructures with a low switching voltage below a few V and a large ME coupling coefficient above 10${\;}^{-5}$ s/m is urgently needed.

## 3. Magneto-electric coupling in artificial multiferroic heterostructures

There is a phenomenon of cross-coupling between non-conjugate intensive and extensive variables in single-phase multiferroics, as illustrated in Figure 1. A similar cross-coupling phenomenon occurs in artificial multiferroic heterostructures, where the magnetic properties can be controlled by an electric field, thus exhibiting ME coupling effects. The mechanisms underlying ME coupling effects in artificial multiferroic heterostructures can be broadly classified into five categories as illustrated in Figure 2: (1) magnetoelastic effect, (2) charge modulation effect, (3) exchange coupling effect, (4) ionic modulation effect, and (5) bonding modulation effect. This section will commence with a concise overview of the mechanisms of ME coupling, followed by an examination of the theoretical and experimental advances in ME effects attributed to the magnetoelastic effect, which is the most predominant mechanism in artificial multiferroic heterostructures.

### 3.1. Mechanisms of magneto-electric coupling

#### 3.1.1. Magnetoelastic coupling effect

**Figure 3.**
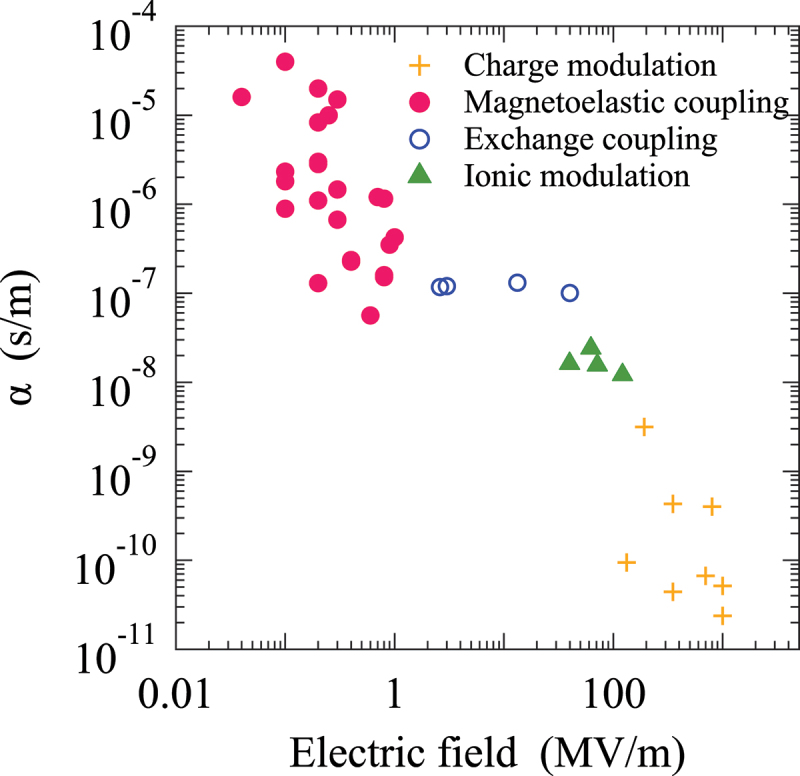
Magneto-electric coupling coefficients in artificial multiferroic heterostructures with various mechanisms.

FE materials exhibit a piezoelectric effect, whereby the application of an electric field to the FE component of an FM/FE heterostructure induces inverse piezoelectric strain. This strain is transferred to the FM layer across the interface, allowing magnetoelastic coupling to modulate the magnetic properties such as magnetization and magnetic anisotropy. The magnetoelastic coupling effect has been observed to occur even at a distance of several hundreds nm from the interface, which is a significantly larger distance than that of other mechanisms. In general, the strength of the ME coupling is quantified by the converse ME coupling coefficient, denoted by α, which is given by the expression: $\alpha =\mu_{0}\mathrm{\partial M}/\mathrm{\partial E}$, where $\mu_{0}$ is the magnetic permeability of vacuum, M is the magnetization, and E is the electric field. This value represents the extent to which magnetization can be modulated by a unit electric field. As illustrated in Figure 3, the ME coefficient is considerably larger than those resulting from other effects. Nevertheless, the magnetoelastic coupling is subject to a limitation in terms of the speed at which magnetic properties respond to an electric field. In order to induce magnetization switching by the magnetoelastic coupling, two key parameters must be considered: the distortion-inducing time of the ferroelectric and the switching time of the magnetization due to Larmor precession [66]. The distortion-inducing time is contingent upon the strength of the electric field applied, with a range of 0.1 μs to 100 s. Conversely, the Larmor precession time is on the order of a few nsec. Therefore, the distortion-inducing time represents the rate-limiting parameter in this mechanism. Nevertheless, the velocity of the FE propagation can be controlled by changing the strength of an electric field over 5 orders of magnitude [32], which is very encouraging to achieve fast switching of the magnetic properties of a FM layer via magnetoelastic coupling. Therefore, magnetoelastic coupling mechanism is the most promising to be incorporated in novel ME spintronic devices.

#### 3.1.2. Charge modulation effect

The application of an electric field of sufficient intensity to saturate the FE polarization in a FM/FE heterostructure results in the creation of polarization charges at the interface. The polarization charges attract spin-polarized electrons towards the interface, thereby inducing additional interfacial magnetization [75,76]. This mechanism of electric field effect on magnetization is referred to as the charge modulation effect. The charge modulation effect bears resemblance to the field effect transistor effect observed in semiconductors. The converse ME coupling coefficient is found to be proportional to the spin polarization of the FM material [77], indicating that fully spin-polarized half metals such as Heusler alloys and manganites are promising candidates for high-performance multiferroic heterostructures. However, it is important to note that there is a screening effect of polarization charges by conduction electrons in FM materials [75], which restricts the region where the ME coupling occurs to within the Thomas-Fermi screening length, approximately 0.1 nm. The small screen length limits an increase in the ME coupling coefficient in this mechanism. However, if the interface-sensitive effect dominates the magnetic properties, this mechanism could also give rise to a large ME effect. Furthermore, the two-dimensional interface magnetotransport could be significantly modulated by an electric field.

#### 3.1.3. Exchange coupling effect

The majority of single-phase multiferroic materials, including BiFeO${\;}_{3}$ [16,17] and YMnO${\;}_{3}$ [41], exhibit both AFM and FE orders concurrently. The cross-coupling between these ferroic orders enables the manipulation of AFM sublattice magnetization orientation by an electric field. When a spin-uncompensated G-type AFM sublattice plane of a single-phase multiferroic material is interfaced with a FM material, exchange coupling occurs between the AFM sublattice spins and the FM spins. This allows the orientation of the FM spins, or magnetization, of the FM material to be controlled via electric field-induced switching of the AFM sublattice magnetization orientation of the single-phase multiferroic material [42,44,78,79]. This mechanism of the ME coupling is referred to as the exchange coupling effect, and manifests within the exchange coupled region, which is characterized by an exchange length of a few nm from the interface. ME coupling arising from this mechanism can also be seen in FM material/AFM magnetoelectric material heterostructures such as Cr${\;}_{2}$O${\;}_{3}$/(Co/Pt)${\;}_{3}$ [80–83]. Recently, giant electric field modulation of the switching field has been demonstrated in the Pt/Cr${\;}_{2}$O${\;}_{3}$/Pt heterostructure, where a boundary magnetization at the Pt/Cr${\;}_{2}$O${\;}_{3}$ coupled to the bulk ME AFM Cr${\;}_{2}$O${\;}_{3}$ can be reversed by an electric field [84]. The efficiency of the switching field becomes 50 times greater than that of conventional FM layer/dielectric oxide interfaces. A deterministic 180${\;}^{\circ }$ electric field reversal of the boundary magnetization can also be achieved, although an additional bias magnetic field is required to form a monostable state. Such naturally created FM/AFM interfaces, due to the broken spatial inversion symmetry at the interface, will be a promising way to achieve a large ME coupling effect.

#### 3.1.4. Ionic modulation effect

The application of an electric field to a FE component of a magnetic/FE heterostructure results in the migration of oxygen ions towards the interface [45,46,85–87]. This process leads to the oxidation of magnetic materials and the modulation of their magnetic properties as demonstrated in Co/GaO${\;}_{x}$ interfaces. Additionally, H${\;}_{2}$O molecules in the atmosphere may undergo decomposition, resulting in the formation of hydrogen (H${\;}^{+}$) and oxygen (O${\;}^{2-}$) ions. These ions are driven by an electric field and facilitate the reduction of oxides in close proximity to the interface [88]. The ME mechanism associated with the electric field-induced redox effects on the magnetic properties of the heterostructures is referred to as the ionic modulation effect. The characteristic length scale associated with the ionic modulation effect is determined by the length scale at which the redox effect occurs, which is typically on the order of 10 nm. Similar ionic modulated effects could occur at the magnetic material/ionic liquid interface, although a change in the number of unpaired d electrons and associated modulation of the magnetic properties by an electric field has been reported at magnetic material/ionic liquid interfaces such as FePt (FePd) or La${\;}_{1-x}$Sr${\;}_{x}$MnO${\;}_{3}$/ionic liquid interfaces [47,71]. While the ionic modulation effect is significant in terms of electric field variation of magnetic properties, the time scale of this effect is on the order of seconds, which will be a serious obstacle to incorporating it into a practical electronic device.

#### 3.1.5. Bonding modulation effect

**Figure 4.**
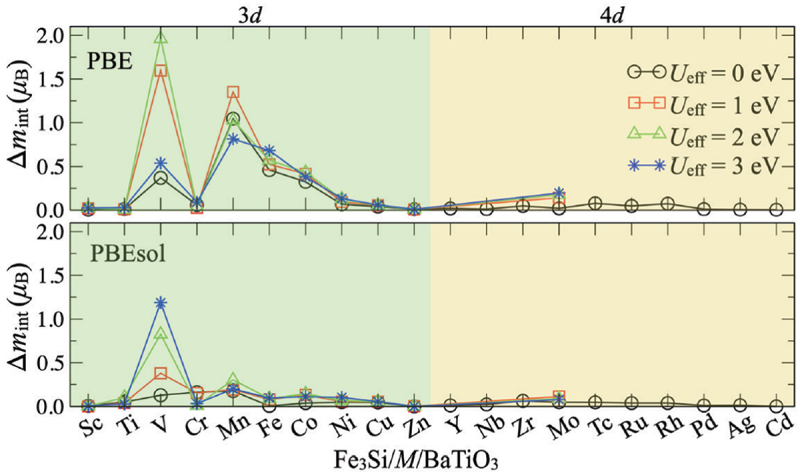
Interfacial magnetic moment upon the polarization reversal in Fe_3_Si/M/BaTiO_3_ heterostructures, where M is the inserted element. Reprinted with permission from [91]. Copyright 2021 the Authors. Phys. Rev. Applied published by American Physical Society.

In an artificial multiferroic heterostructure comprising a displacive type of FE material, such as BaTiO${\;}_{3}$, the reversal of the electric field induces the motion of cations within the ferroelectric, thereby modulating the orbital hybridization (chemical bonding) of the constituent ions at the interface [19,48,89,90]. This results in the additional magnetic moment in the cations in proximity to the interface, contingent upon the polarity of the applied electric field. The ME mechanism associated with the modulation of orbital hybridization is referred to as the bonding modulation effect. Given that this type of ME effect arises from chemical bonding in close proximity to the interface, the characteristic length is on the order of less than 1 nm. This short characteristic length indicates that the use of 2-dimensional interface-related phenomena is required to induce a large electric field modulation effect on the magnetic properties as discussed in the charge modulation mechanism. This will encourage us to discover novel phenomena to integrate this effect in device applications. Also, the ME coefficient is expected to be significantly affected by the insertion of a different atomic layer at the FM/FE interface. For instance, Costa-Amaral *et al*. reported on the atomic layer insertion effect on the ME effect of Fe${\;}_{3}$Si/M/BaTiO${\;}_{3}$ heterostructures, demonstrating a pronounced inserted element dependence of the ME coefficient (Figure 4) [91].

From the discussions of the five predominant mechanisms of the ME coupling effect in artificial mutiferroic heterostructures, we now realize that the most promising approach to develop novel device applications based on the ME effect will be to exploit the magnetoelastic coupling effect: huge ME coupling coefficients above 10${\;}^{-5}$ s/m as shown in Figure 3 and fast switching speed on the order of ns by exploiting the FE domain wall motion in a sub-micron artificial mutiferroic heterostructure. From this point of view, in the following we will focus on the artificial mutiferroic heterostructures based on the magnetoelastic coupling effect.

### 3.2. Theoretical description of ME effect due to magnetoelastic coupling

As stated before, the modulation of magnetic anisotropy by magnetoelastic coupling is a consequence of the exertion of strain on a magnetic material. The variation in magnetic anisotropy can be described in classical terms as a change in free energy due to strain exerted on the material, as expressed as follows [6,28,92,93]:

(1) $$\begin{array}{rl} & \mathrm{\Delta F}≅K_{1}m_{1}^{2}m_{2}^{2}+\left(K_{1}+\frac{B_{1}^{2}}{2c_{11}}-\frac{B_{2}^{2}}{2c_{44}}\right)(m_{1}^{2}+m_{2}^{2})m_{3}^{2}\text{\textbackslash{}break}\;\;\;\;\;\;\;\;\;\;\;\;\;\;\;+\;K_{2}m_{1}^{2}m_{2}^{2}m_{3}^{2}+B_{1}(u_{1}m_{1}^{2}+u_{2}m_{2}^{2})+B_{2}u_{6}m_{1}m_{2}\text{\textbackslash{}break}\;\;\;\;\;\;-B_{1}\left[\frac{B_{1}}{6c_{11}}+\frac{c_{12}}{c_{11}}(u_{1}+u_{2})\right]m_{3}^{2}\\ & +\frac{K_{s}}{t}m_{3}^{2}\text{\textbackslash{}break}\;\;\;\;\;\;\;\;\;\;\;\;+\frac{1}{2}\mu_{0}M_{S}^{2}(N_{1}m_{1}^{2}+N_{2}m_{2}^{2}+N_{3}m_{3}^{2}), \end{array}$$

where $m_{i}$ (i= 1, 2, 3) are the direction cosines of the unit vector $m=M/M_{s}$ (M and $M_{s}$ are the magnetization vector and the saturation magnetization), $K_{1}$ and $K_{2}$ are the magnetocrystalline anisotropy constants of fourth and sixth order at constant strains ***u***, t is the FM layer thickness, $K_{s}$ is the interface anisotropy constant, $N_{i}$ are the diagonal components of the tensor of demagnetizing factors [94], $B_{1}$ and $B_{2}$ are the magnetoelastic coefficients [95], and $c_{11}$, $c_{12}$, and $c_{44}$ are the elastic stiffnesses at fixed ***M***. By means of this equation, the numerical minimization of the free energy allows us to determine the most stable magnetization orientation as a function of the strain u. In an artificial multiferroic heterostructure, the application of strain to the FM layer is achieved through the transfer of strain due to the inverse piezoelectric effect of the FE substrate by an electric field, resulting in a variation of the magnetization orientation. This classical description offers a qualitative account of the ME effect observed in artificial multiferroic heterostructures, whereby magnetoelastic coupling plays a role. However, it does not provide guidance on the selection of materials and interfaces that would be optimal for fabricating a new artificial multiferroic heterostructure with a large ME coefficient. Consequently, a microscopic theoretical description is required.

**Figure 5.**
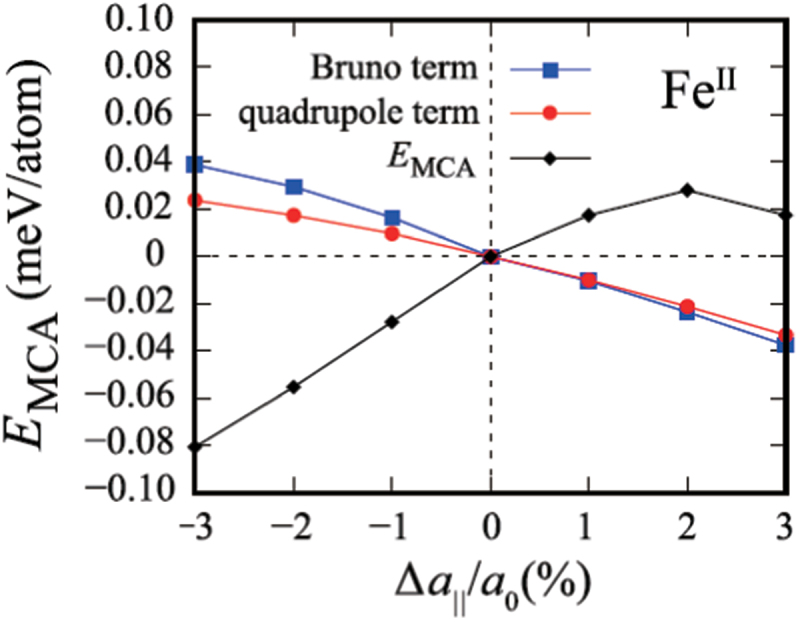
Atom-resolved $E_{MCA}$ and the contributions from the Bruno and quadrupole terms of Fe${\;}_{3}$Si as a function of strain $\Delta a_{∥}/a_{0}$. Reprinted with permission from [97]. Copyright 2023 IOP Publishing.

In accordance with the tenets of second-order perturbation theory, the magnetocrystalline anisotropy energy ($E_{MCA}$) can be expressed as a sum of the four distinct energy contributions associated with the virtual excitation processes between the occupied and unoccupied states.

(2) $$E_{MCA}=E_{MCA}^{↑↑}+E_{MCA}^{↓↓}+E_{MCA}^{↑↓}+E_{MCA}^{↓↑},$$

where $E_{MCA}^{\sigma_{1}\sigma 2}$ denotes the magnetocrystalline anisotropy energy due to the virtual excitation from the occupied spin $\sigma_{1}$ state to the unoccupied $\sigma_{2}$ state. Of the four terms, $E_{MCA}^{↓↓}$ is associated with the orbital moment anisotropy, or the difference in orbital angular momentum along different axes, represented by the $\Delta m_{orb}$. $E_{MCA}^{↑↓}$ is linked to the spin moment, or the magnetic moment represented by $m_{D}$ as a result of the quadrupole moment of the electron density distribution. The former is referred to as the Bruno term, while the latter is designated as the quadrupole term, as expressed in the following equations.

(3) $$E_{MCA}^{↓↓}\approx -\frac{1}{4\mu_{B}}\sum \xi \Delta m_{orb},$$

(4) $$E_{MCA}^{↑↓}\approx -\frac{3}{8\mu_{B}}\sum \frac{\xi^{2}}{\Delta \varepsilon_{ex}}m_{D},$$

where ξ is the spin-orbit constant and $\Delta \varepsilon_{ex}$ is the averaged exchange splitting energy. Given that orbital moments can be estimated through X-ray magnetic circular dichroism (XMCD) experiments, Eq. (4) is employed to elucidate the genesis of magnetic anisotropy. However, it should be noted that consideration of the other excitation processes is also necessary to gain a comprehensive understanding of both the mechanism of the magnetocrystalline anisotropy and its effect of strain transfer, as discussed by van der Laan in greater detail [96]. Indeed, Yatmeidhy *et al*. conducted first-principles electronic structure calculations of the total $E_{MCA}$ and the Bruno term and quadrupole-term contributions as a function of in-plane lattice strain. The strain dependence of the Bruno term and the quadrupole-term contributions to the total $E_{MCA}$ exhibits a distinct behavior from that of the total $E_{MCA}$, as illustrated in Figure 5 [97]. This unequivocally demonstrates the necessity for a comprehensive and meticulous examination of the strain transfer effect on total $E_{MCA}$ and magnetic anisotropy in artificial multiferroic heterostructures.

### 3.3. Advances in large ME effect due to magnetoelastic coupling

As illustrated in Figure 3, markedly large converse ME coupling coefficients are evident in artificial multiferroic heterostructures wherein the ME mechanism is attributed to the magnetoelastic coupling effect. A variety of combinations of FM and FE materials are employed in the construction of these heterostructures as shown in Table 1. The most commonly utilized FE materials are BaTiO${\;}_{3}$ (BTO), PbZr${\;}_{1-x}$Ti${\;}_{x}$O${\;}_{3}$ (PZT), and Pb(Mg${\;}_{1/3}$Nb${\;}_{2/3}$)O${\;}_{3}$-PbTiO${\;}_{3}$ (PMN-PT). In the context of FM materials, 3d transition metals such as Fe, Co, and Ni are employed, whereas amorphous FM metals (CoFeB), Heusller alloys (Co${\;}_{2}$FeSi), chemically ordered alloys (FeRh, SmCo, Co${\;}_{3}$Mn), magnetostriction FM materials (FeGa), magnetic oxides (Fe${\;}_{3}$O${\;}_{4}$), and magnetic multilayers (Cu/Ni, Co/Pd, Pt/Co/Ta) are used. The selection of different material combinations has resulted in the observation of a wide range of ME coefficients. In this section, we present the recent findings on the large converse ME coupling coefficient observed in Co${\;}_{2}$FeSi/PMN-PT and Co${\;}_{2}$FeSi/BaTiO${\;}_{3}$ heterostructures, along with an analysis of the underlying mechanisms.

**Figure 6.**
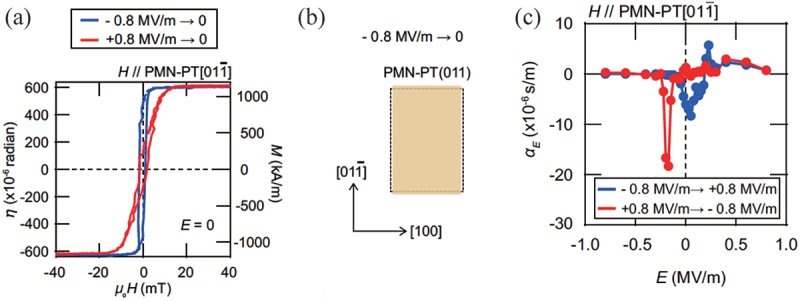
(a) Magnetization curves of Co${\;}_{2}$FeSi/PMN-PT(011) along PMN-PT[01$\overset{ˉ}{1}$] directions after positively and negatively poling the PMN-PT. (b) a schematic diagram of a change in the PMN-PT(011) plane after negative poling. (c) ME coefficient while negative and positive poling processes. Reprinted with permission from [26]. Copyright 2022 the Authors. NPG Asia Mater. published by Springer Nature.

**Figure 7.**
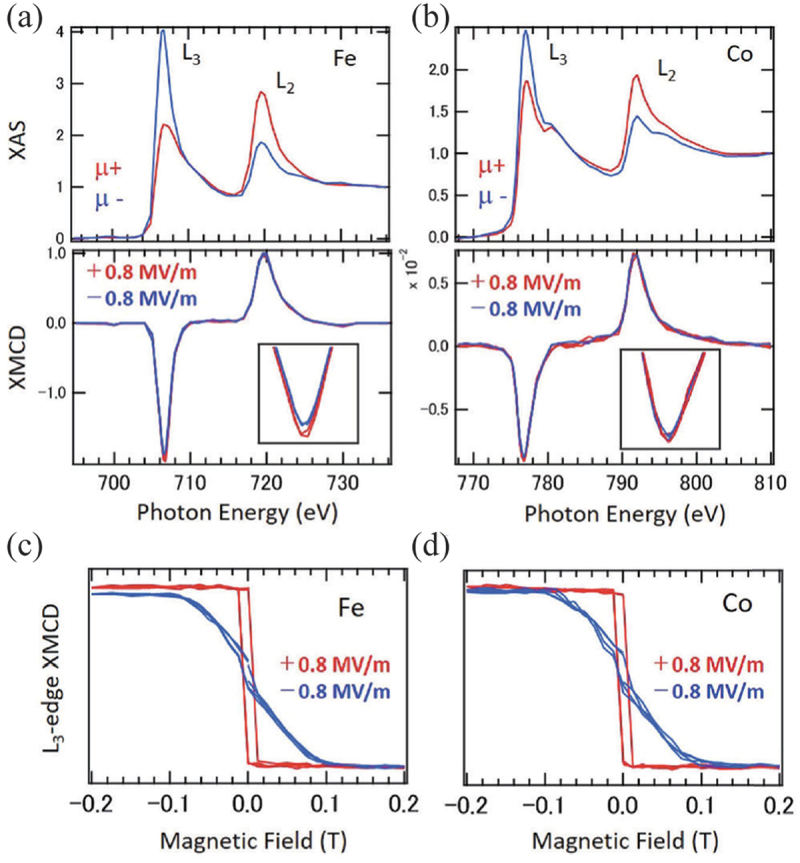
X-ray absorption and XMCD spectra of (a) Fe and (b) Co sites. (c) (d) Magnetic field dependence of L${\;}_{3}$ edge XMCD for Fe and Co site, respectively. Reprinted with permission from [99]. Copyright 2024 the Authors. NPG Asia Mater. published by Springer Nature.

Recently, Fujii *et al*. reported a significant ME coefficient of 1.2–1.8$\times 10^{-5}$ s/m in FM Heusler alloy Co${\;}_{2}$FeSi/ferroelectric PMN-PT (011) heterostructures, as shown in Figure 6 [26]. When the PMN-PT substrate is poled positively, the magnetic easy axis is oriented along the PMN-PT[01$\overset{ˉ}{1}$] direction. Conversely, when the substrate is poled negatively, the magnetic easy axis is oriented along the PMN-PT [98] direction. This demonstrates that switching the electric field polarity can control the magnetic easy axis at the remanent electric field state due to transfer of lattice distortion of PMN-PT as illustrated in Figure 6(b), indicating that the magnetization orientation can be non-volatilely controlled. Also, the Co${\;}_{2}$FeSi/PMN-PT (011) heterostructure exhibits a substantial ME coefficient, as illustrated in Figure 6(c). The origin of this considerable ME coefficient is further examined by Okabayashi *et al*. through element-specific XMCD measurements, as depicted in Figure 7 [99]. The XMCD spectra of the Fe site at the $L_{3}$ edge demonstrate varying magnitudes for positive and negative poling, whereas those of the Co site exhibit no electric field polarity dependence. The difference in the XMCD spectra at the Fe site after poling is equivalent to a change in the orbital magnetic moment of Fe by 0.01 $\mu_{B}$, as estimated by the XMCD sum rule. These findings suggest that the switching of the magnetic anisotropy is linked to the electric field-induced alteration in the orbital magnetic moments of Fe in Co${\;}_{2}$FeSi.

**Figure 8.**
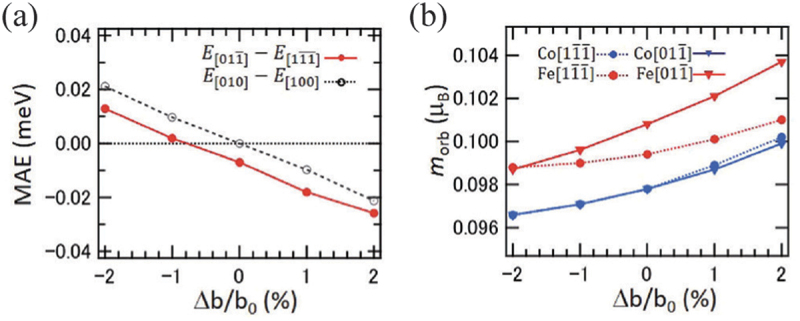
(a) Magnetic anisotropy energy and (b) orbital magnetic moment as a function of in b-axis lattice strain. Reprinted with permission from [99]. Copyright 2024 the Authors. NPG Asia Mater. published by Springer Nature.

It is also noteworthy that the Co${\;}_{2}$FeSi film exhibits a (422) crystal orientation on PMN-PT(011) with an $L2_{1}$ ordered structure. For this crystal orientation, first-principles density functional theory calculations demonstrated there is a discernible correlation between the magnetic anisotropy, orbital magnetic moment, and lattice strain along the Fe [01$\overset{ˉ}{1}$] direction. As shown in Figure 8, the magnetic anisotropy energy diminishes with tensile strain along the b-axis. Additionally, the orbital magnetic moments of Fe show a crystal orientation dependence, whereas the Co orbital magnetic moments exhibit no orientation dependence on the (422) plane, which agrees with the experimental results [99]. The results clearly indicate that the selection of the lattice plane is of critical importance for achieving a large ME coefficient in multiferroic heterostructures. A similar large ME coefficient has been reported in magnetostrictive FeGa/PMN-PT heterostructures, where a ME coefficient of approximately $2.0\times 10^{-5}$ s/m was achieved [27,100], although the microscopic mechanism is different.

The importance of the crystal structure is also pointed out to achieve a large converse ME coefficient. Murakami *et al*. investigated the ME effect of metastable Co${\;}_{3}$Mn/Fe/PMN-PT(001) heterostructures with different crystallized Co${\;}_{3}$Mn: bcc, bcc+fcc/hcp, and fcc/hcp structures [98]. A multiferroic heterostructure with bcc Co${\;}_{3}$Mn shows 4.7–8.3$\times 10^{-6}$ s/m, while that with fcc/hcp Co${\;}_{3}$Mn shows no ME effects. The authors attribute this to the presence or absence of magnetic anisotropy, since bcc Co${\;}_{3}$Mn exhibits uniaxial magnetic anisotropy with an anisotropy constant of 1.5$\times 10^{4}$ erg/cm${\;}^{3}$ in contrast to no magnetic anisotropy in fcc/hcp Co${\;}_{3}$Mn heterostructures. First-principles calculation of the magnetic anisotropy also ensures that the magnetic anisotropy and its in-plane strain dependence are larger for bcc Co${\;}_{3}$Mn than for fcc Co${\;}_{3}$Mn, in good agreement with experimental results. It should also be noted that the magnetic anisotropy is found to be proportional to $\Delta m_{orb}$ in Eq. 4, following the Bruno relation, in contrast to the Co${\;}_{2}$FeSi/PMN-PT(011) described above.

**Figure 9.**
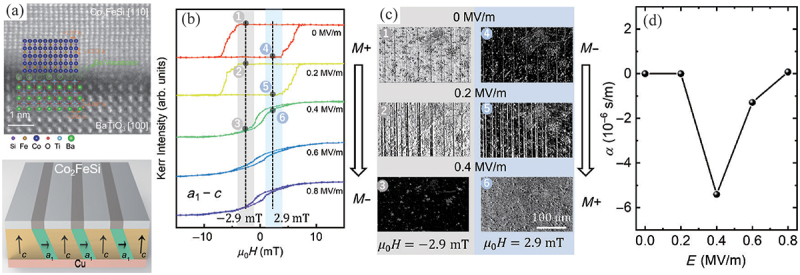
(a) a TEM image of Co${\;}_{2}$FeSi/BaTiO${\;}_{3}$ heterostructure and a schematic FM and FE domain structures. (b) Magnetization curves in different electric field and (c) Magnetooptical Kerr images in different electric field. (d) The ME coefficient as a function of electric field. Reprinted with permission from [104]. Copyright 2023 American Physical Society.

Another promising avenue for enhancing a ME coefficient is the control of FE domain structures. A representative example of electric field control of FE domain structures can be observed in BaTiO${\;}_{3}$. The crystal structure of BaTiO${\;}_{3}$ is tetragonal at room temperature, which gives rise to three distinct domain structures: the c-domain, the $a_{1}$-c-domain, and the $a_{2}$-c-domain. The c-domain exhibits a square lattice on the surface, whereas the $a_{1}$-c and $a_{2}$-c domains are alternately square and rectangular stripe domain structures. The epitaxial growth of a FM material on a BaTiO${\;}_{3}$ substrate results in the transfer of different strains to the FM material, giving rise to a distinct magnetic anisotropy, which is dependent on the FE domain in which the FM material is grown. This type of pattern transfer from FE to FM materials was confirmed by previous reports by Lahtinen *et al*. [101–103]. Furthermore, the magnetic anisotropy can be controlled by an electric field, given that the FE domain can be switched by an electric field. A clear demonstration of the ME effect due to such domain switching has been reported by Hu et al. [104]. As illustrated in Figure 9, clear magnetic domain patterns resulting from strain transfer have been observed in an epitaxial Co${\;}_{2}$FeSi/BaTiO${\;}_{3}$ heterostructure. When an electric field is applied perpendicular to the $a_{1}$-c-domain stripe, the square-shaped magnetic hysteresis curve undergoes a transformation to a slanted hysteresis curve, reflecting a switching of magnetic anisotropy. Additionally, a relatively large ME coefficient, approximately $5.8\times 10^{-6}$ s/m, can be achieved through the strain transfer-induced switching of magnetic anisotropy resulting from FE domain switching (Figure 9d). A significant domain switching-induced converse ME coefficient of 1.6$\times 10^{-5}$ s/m also has been reported in FeRh/BaTiO${\;}_{3}$ heterostructures, wherein a magnetic phase transition between the FM and AFM phases of FeRh is triggered by strain transfer [21]. It is also demonstrated that the perpendicular magnetic anisotropy in a [Cu/Ni] multilayer on BaTiO${\;}_{3}$ can be switched from out-of-plane to in-plane by an electric field with compressive strain transfer [28]. These results clearly indicate that not only the piezostrain, but also the ferroelastic strain induced by the domain switching is crucial for achieving a large ME coefficient.

## 4. Applications of artificial multiferroic heterostructures

The ability to manipulate various magnetic properties via an electric field, enabled by multiferroic heterostructures, offers a promising avenue for the development of novel low-power spintronic and magnonic devices. Since an energy dissipation necessary to control the magnetic properties of artificial multiferroic heterostructures is the charging energy of the FE layer, this can be estimated to be 100–500 μJ/cm${\;}^{2}$ per switching, assuming a thickness of the FE layer of 100–200 nm, a saturation polarization of 50 μC/cm${\;}^{2}$, and the switching voltage of 1–5 V. The value is a factor of 10–100 lower than that required by spin transfer torque ∼3-4 mJ/cm${\;}^{2}$ [6,16]. Therefore, artificial multiferroic heterostructures offer great promise to be incorporated into low-power spintronic device applications. In this section, we present an overview of recent reports on the electric field control of various magnetic and superconducting properties using artificial multiferroic heterostructures.

### 4.1. Perpendicular magnetic anisotropy

Control of perpendicular magnetic anisotropy (PMA) is one of the most critical issues for spintronic and magnetic applications such spin random access memory and high density magnetic recording. A traditional technology uses spin transfer torque to switch the perpendicularly oriented magnetization in these devices while electric field-induced strain transfer also could control the PMA in artificial multiferroic heterostructures. Shirahata *et al*. demonstrated purely electric field switching of PMA in [Cu/Ni] multilayers via strain transfer from BaTiO${\;}_{3}$ that exhibits FE a-domain to c-domain siwtching, thereby a large converse ME coupling coefficient of $6\times 10^{-7}$ s/m was achieved [28]. Also, 180${\;}^{\circ }$ magnetization reversal of the perpendicularly oriented magnetization was demonstrated by an electric field pulse with the assistance of a tiny magnetic field. Electric field modulation of the spin and orbital magnetic moments of the Ni layers has been measured using XMCD sum rules, estimating a change in the orbital magnetic moment of 0.01$\mu_{B}$ while no changes in the spin magnetic moment [105]. Therefore, the authors attribute the electric field modulation in PMA to the variation in the orbital magnetic moments induced by strain transfer through an electric field. In a report by Chen *et al*. electric field modulation of PMA was also demonstrated in Pt/Co/Ta thin films using anomalous Hall effect, presenting a large converse ME coefficient reaching $2.1\times 10^{-6}$ s/m in a small bias magnetic field of −20 Oe [106]. The authors discussed the origin of the large ME effect that is associated with the strain mediated interface roughness induced by an electric field.

**Figure 10.**
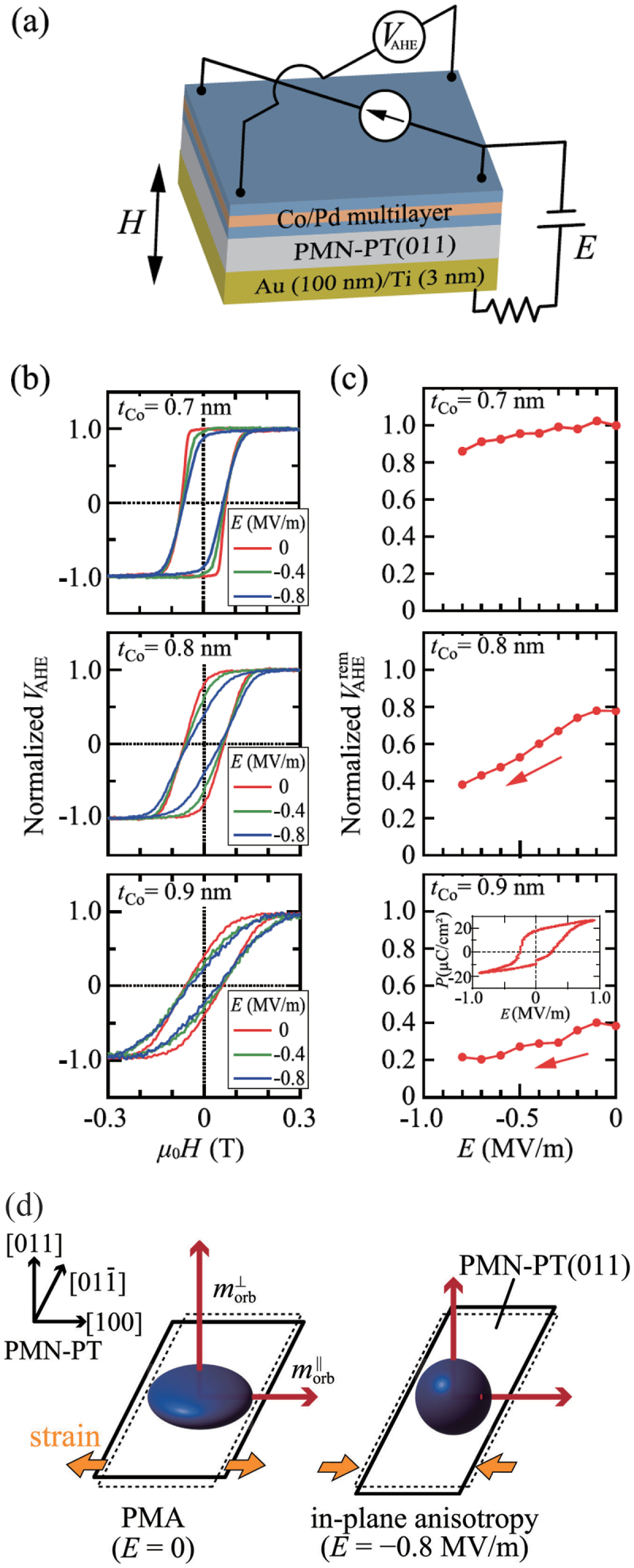
(a) Schematic of [Co/Pd] multilayer/PMN-PT(011) multiferroic heterostructures. (b) Electric field dependent magnetization curves of [Co/Pd] multilayer/PMN-PT(011) with different Co layer thicknesses. (c) Electric field dependence of the normalized anomalous Hall voltage at the remanent state for the samples in (b). (d) Schematic diagrams of the orbital magnetic moments (red arrows) and the corresponding distributions of the Co d orbitals (blue ellipsoid). Reprinted with permission from [29]. Copyright 2023 Elsevier B.V.

Recently, Usami *et al*. also demonstrated electric field manipulation of PMA in [Co/Pd] multilayer/PMN-PT (011) multiferroic heterostructures at the remanent state as shown in Figure 10 [29]. By tuning the Co layer thickness in the range where the PMA changes to in-plane anisotropy, the authors achieved a ME coefficient greater than 1.0$\times 10^{-6}$ s/m. The authors ascribed the electric field induced switching of the PMA to in-plane anisotropy to possible piezostrain induced variation in the symmetry of the Co orbital magnetic moment due to a change in the occupation of the 3d $z^{2}$ orbital, taking into account the mechanism of the PMA in [Co/Pd] multilayers previously reported by XMCD measurements [107]. As shown in Figure 10(d), the Co orbitals composed of Co 3d(xy, $x^{2}-y^{2}$) and 3d(yz, zx) orbitals or the 3d electron distribution has a flattened pancake shape before the application of an electric field, leading to an enhancement of the anisotropic perpendicular orbital magnetic moment. On the contrary, when an electric field is applied, the compressive in-plane piezostrain along PMN-PT [98] increases the electron occupation in the 3d $z^{2}$ orbitals, changing the 3d electron distribution to a spherical-like shape. This spherical electron distribution gives rise to the isotropic orbital magnetic moments and a reduction in the PMA. These results are very promising for developing novel energy-efficient spintronic devices surpassing the current based spintronic technology. 180${\;}^{\circ }$ magnetization switching driven by a pure electric field has also theoretically demonstrated [24,108], further encouraging the incorporation of artificial multiferroic heterostructures into spintronic devices.

### 4.2. Magnetoresistance

Magnetoresistance (MR) is a crucial property for the development of spintronic devices. A variety of mechanisms of MR in magnetic heterostructures have been investigated, including anisotropic magnetoresistance (AMR), giant magnetoresistance (GMR), and tunneling magnetoresistance (TMR). These mechanisms are contingent upon the orientation of the magnetic layers. As previously discussed, the orientation of the magnetic layers can be manipulated by an electric field in multiferroic heterostructures, thereby enabling the potential control of MR through an electric field.

**Figure 11.**
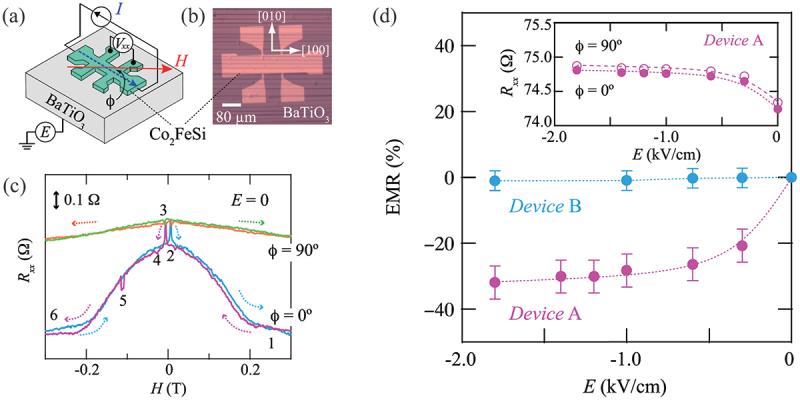
(a) Schematic and (b) optical micrograph of a fabricated Co${\;}_{2}$FeSi Hall-bar device on BaTiO${\;}_{3}$. (c) Room-temperature $R_{\mathrm{xx}}$-H curves of a device for H//[100] of BaTiO${\;}_{3}$ and H⊥[100] of BaTiO${\;}_{3}$ at an E of zero. (d) EMR as a function of electric field for two different samples. Reprinted with permission from [72]. Copyright 2021 American Physical Society.

In a recent study, Yamada *et al*. demonstrated the electric field control of MR in Co${\;}_{2}$FeSi/BaTiO${\;}_{3}$ heterostructures, which exhibited a notable phenomenon due to AMR [72]. As illustrated in Figure 11, epitaxial Co${\;}_{2}$FeSi/BaTiO${\;}_{3}$ heterostructures that are microfabricated into Hall-bar device structures demonstrate a discernible AMR, presumably due to coherent magnetization rotation. The AMR is defined as the difference in the resistances $R_{\mathrm{xx}}$ for magnetization orientations parallel and perpendicular to the current direction. The AMR is clearly observed to vary as a function of the electric field as shown in Figure 11(d), where the term ‘electromagnetoresistance’ (EMR) is defined as the ratio of the change in MR to the initial MR. It is evident that the magnitude of EMR is markedly enhanced with an increase in electric field strength, reaching a maximum of approximately 32%. Furthermore, the authors ascribe the observed sample dependence (Device A with an a-c domain structure and Device B without it) to the motion of the FE a-c domain wall. In general, the AMR is given by the equation Δρ/ρ=γ(α−1), where $\Delta \rho =(\rho_{//}-\rho_{⊥})$, $\alpha =\rho_{↓}/\rho_{↑}$, $\gamma =(\lambda /E_{\mathrm{ex}}{)}^{2}$, $\rho_{↑}(↓)$ is the resistivity for the majority (↑) and minority (↓) spin channels, λ is the spin-orbit coupling constant, and $E_{\mathrm{ex}}$ is the exchange interaction energy [109]. This suggests that the observed enhancement of the EMR is possibly due to the modulation of λ and $E_{\mathrm{ex}}$ resulting from lattice distortion via strain transfer arising from the a-c domain wall motion in BaTiO${\;}_{3}$. Another theoretical account of the EMR effect in Co${\;}_{2}$FeSi/BaTiO${\;}_{3}$ heterostructures has also been given by Tsuna *et al*. from first-principles calculations [110]. The authors discuss the 3$d_{\mathrm{yz}}$ density of states for the FeSi/TiO${\;}_{2}$ and CoCo/TiO${\;}_{2}$ interfaces with TiO${\;}_{2}$ termination and show that the minority spin density of states for the FeSi/TiO${\;}_{2}$ interface increases by electric field-induced switching of the FE domain structure from the a to the c domains. This could modify the AMR by changing the $\alpha =\rho_{↓}/\rho_{↑}$. The electric field modulation of the MR has also been reported in a Co/Cu/Fe trilayer/BaTiO${\;}_{3}$ structure [73]. Very recently, electric field modulation of magnetoresistance (TMR) ratio by 55% was demonstrated in MgO-based tunnel TMR structure, which is quite encouraging in practical device applications of artificial multiferroic heterostructures [111–113].

### 4.3. Interlayer exchange coupling

**Figure 12.**
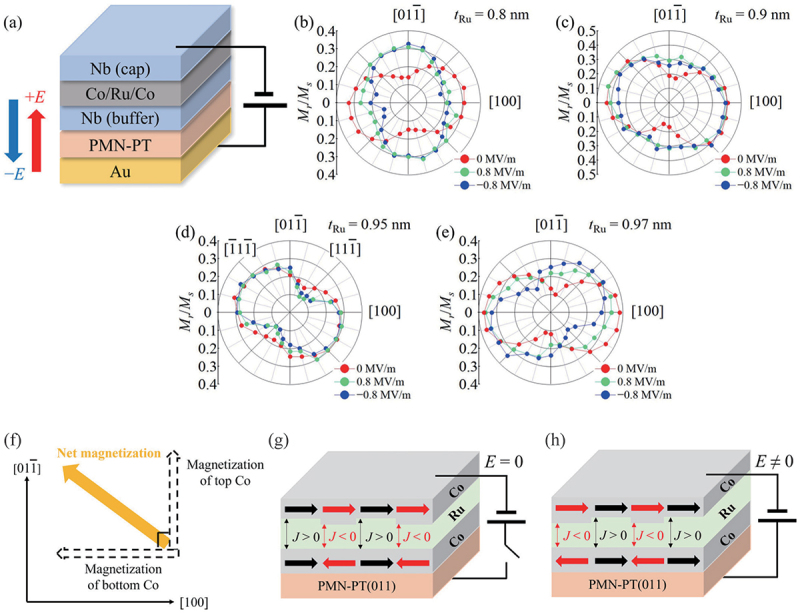
(a) Schematic illustration of a Co/Ru/Co SAF/PMN-PT(011) sample structure for electric field measurements, where an electric field is applied along the [011] direction of the PMN-PT. (b)-(e) In-plane angular dependence of the remanent magnetization ($M_{r}$) normalized by the saturation magnetization ($M_{s}$) for $t_{Ru}=0.8$, 0.9, 0.95, and 0.97 nm, respectively. The red curve shows the data at E=0 MV/m and the green and blue curves show the data at E=0.8 and 0.8 MV/m, respectively. (f) Magnetization configuration near the FM-AFM coupling boundary. (g) Coexistence of FM and AFM coupling and (h) its electric field-induced local modulation. Reprinted with permission from [36]. Copyright 2023 AIP Publishing.

In an FM/nonmagnetic (NM)/FM trilayer, the interlayer exchange coupling strength between the two FM layers shows an oscillatory variation as a function of the thickness of the NM layer [114]. When the two FM layers couple antiferromagnetically, the trilayer is designated a synthetic antiferromagnet, which exhibits a net magnetization of zero. Recently, synthetic antiferromagnets have garnered significant interest for potential applications in AFM spintronics and the control of interlayer exchange coupling represents a crucial area of investigation. To demonstrate the control of the interlayer exchange coupling by an electric field, Wang *et al*. investigated the influence of an electric field on the magnetization process of FeCoB/Ru/FeCoB/PMN-PT(011). Their findings indicate that the interlayer exchange coupling could be modulated via strain transfer induced by the inverse piezoelectric effect of PMN-PT [115]. A comparable effect has been reported by Peng *et al*. [116] Hisada *et al*. have recently investigated the electric field dependence of the magnetic anisotropy of a synthetic ferrimagnet Co/Ru/Co/PMN-PT(011) with different Co layer thicknesses [36]. The authors demonstrated that in both the AFM and FM coupled Ru thickness regimes, the magnetic anisotropy undergoes a 90${\;}^{\circ }$ switching when the polarity of the electric field applied is reversed. In contrast, in the intermediate region between the FM and AFM couplings, the magnetic easy axis is tilted by 45${\;}^{\circ }$, and the electric field polarity dependence on the magnetic anisotropy is no longer observed as shown in Figure 12. The authors attribute this phenomenon to the existence of a mixed FM and AFM coupled region as shown in Figure 12(g). When the interlayer coupling strength is small, a possible inhomogeneity of the interlayer thickness causes a mixing of the FM and AFM coupled regions, so that the competition between the FM and AFM coupled regions leads to a 90${\;}^{\circ }$ magnetization orientation of the two Co layers due to biquadratic coupling. This leads to a 45${\;}^{\circ }$ tiling of the net magnetization near the boundary between the FM and AFM coupled regions (Figure 12(f)). In this mixed-coupled regime, the application of an electric field could switch the FM and AFM coupled regions to each other, leaving the orientation of the net magnetization unchanged, as shown in Figure 12(g,h). These findings may provide a basis for the development of a novel device structure that would permit the electric field switching of the interlayer coupling between synthetic AFM and FM orientations in multiferroic heterostructures.

### 4.4. Spin wave propagation

**Figure 13.**
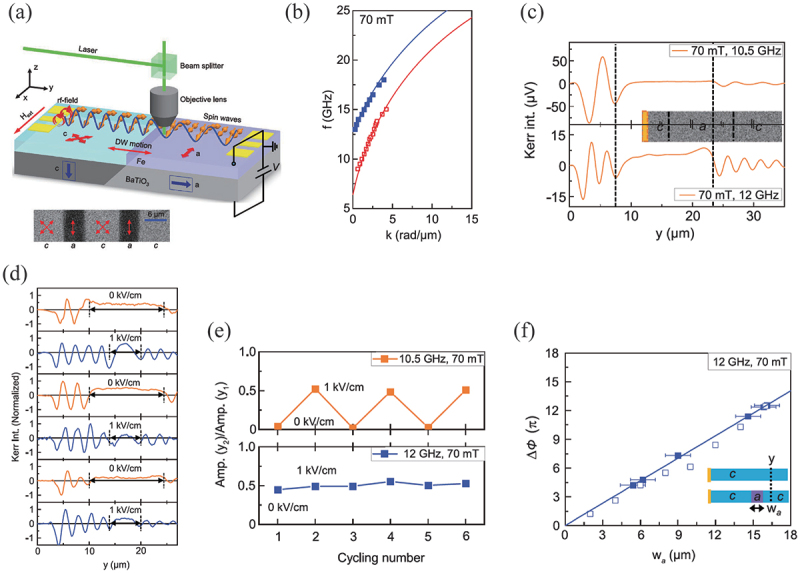
(a) Schematic of spin-wave transmission in strain-coupled domains of a Fe/BaTiO${\;}_{3}$ heterostructure. (b) Spin-wave dispersion relations for an Fe film on top of FE a and c domains in a magnetic bias field of 70 mT. (c) Spin-wave transport across a c−a−c domain structure for the excitation frequency of 10.5 and 12.0 GHz. (d) Spin-wave profiles recorded after repeated switching of the c−a−c domain structure. (e) Variation of the spin-wave amplitude at 10.5 and 12.0 GHz when the electric field is turned on and off repeatedly. (f) Spin-wave phase change Δϕ at y=25 μm as a function of a-domain width at 12.0 GHz and 70 mT. Reprinted with permission from [35]. Copyright 2021 the Authors. Adv. Mater. published by Wiley-vch GmbH.

The control of spin waves in FM materials offers significant potential for device applications, particularly in the field of magnonics. This enables the creation of innovative logic devices that utilize the interference of spin waves [117]. The generation and control of spin waves by an electric field allows for the realization of magnonic devices with minimal power consumption, as the propagation of spin waves in magnetic materials is a less energy dissipative process. Qin *et al*. have reported the control of spin wave propagation in Fe/BaTiO${\;}_{3}$ multiferroic heterostructures [35]. As illustrated in Figure 13(a), the magnetic easy axis of Fe on c-domains of BaTiO${\;}_{3}$ is aligned with the ⟨110⟩ direction, whereas on a-domains, the magnetic easy axis is oriented along the ⟨010⟩ direction of BaTiO${\;}_{3}$. The distinct two magnetic anisotropies give rise to different dispersion relations on the c- and a-domains as shown in Figure 13(b). Upon the excitation of spin waves in Fe on the c-domain of BaTiO${\;}_{3}$, the spin waves propagate and ultimately reach a c-a domain boundary. If the frequency of the excited spin wave exceeds 12 GHz, the spin wave can propagate across the c-a domain boundary due to the presence of a spin wave dispersion on the a-domain at that energy. However, if the spin wave energy is below the minimum energy of the spin wave branch on the a domain, transmission across the c-a domain boundary is not possible as shown in Figure 13(c). Since the c-a domain boundary of BaTiO${\;}_{3}$ can be driven by an electric field [32], the distance of the spin wave propagation can be controlled by applying an electric field as shown in Figure 13(d). This also means that in Fe/BaTiO${\;}_{3}$ heterostructures the electric field can be used to switch the spin wave amplitude or the spin wave propagation at a given point. Indeed, the authors have demonstrated the ability to switch the propagation of spin waves using an electric field, as illustrated in Figure 13(e), by an electric field at 10.5 GHz. Moreover, the phase of spin waves transmitting across a c-a-c domain boundary can be modulated by modifying the width of the a-domain, as the wave numbers of the spin wave on the c-domain and the a-domain are distinct at a given frequency (Figure 13(f)). These demonstrations could provide a novel approach to energy-efficient control of spin wave propagation for magnonic applications. Recently, the electric field control of the spin wave Doppler shift was also theoretically investigated by Hu *et al*. using an Fe/BaTiO${\;}_{3}$ heterostructure with micromagnetics simulation [118]. When spin waves are reflected or transmitted at the boundary of different magnetic anisotropy due to the a-c domain boundary of BaTiO${\;}_{3}$, the red shift or blue shift of the spin wave frequency can be seen upon moving the magnetic anisotropy boundary. Therefore, a comprehensive analysis of the Doppler shift could be used to probe the velocity of the a-c domain boundary of FE driven by an electric field.

### 4.5. Spin damping

**Figure 14.**
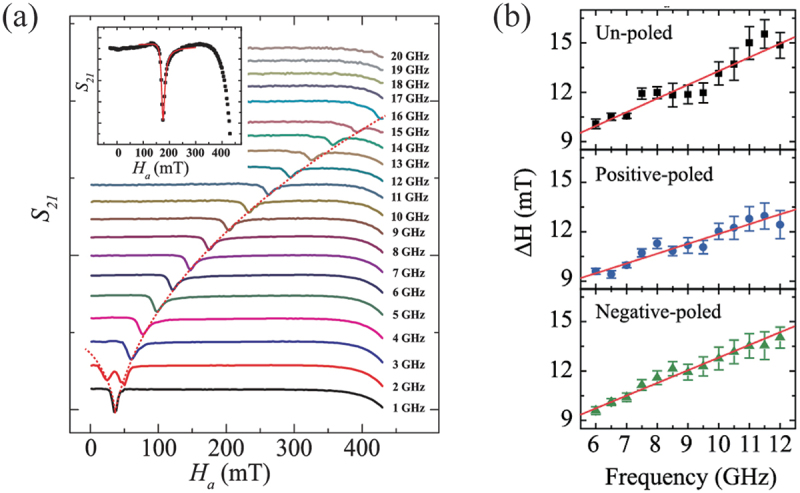
(a) Magnetic field dependent FMR spectra recorded at different frequencies. The inset shows a fit with the asymmetric Lorentzian line shape to the experimental data recorded at 8 GHz. FMR linewidth versus frequency plots of the unpoled and poled states when the applied magnetic field is along the magnetically (b) easy axis and (b) hard axis. Reprinted with permission from [119]. Copyright 2023 IOP Publishing.

A low Gilbert damping constant is a crucial parameter of FM materials, as it enables not only long-range propagation of spin waves but also low-power switching of magnetization by spin transfer torque [1]. The characterization of the Gilbert damping constant, α, of a FM material is commonly achieved through the use of ferromagnetic resonance (FMR). In general, the FMR linewidth is given by the following equation: $\mathrm{\Delta H}(f_{r})=\Delta H_{0}+2\pi (2m_{0}c/\mathrm{ge})\alpha f_{r}$, where $\Delta H_{0}$ is a measure of the extrinsic contribution to the linewidth due to inhomogeneity of the film, g is the Land*é* g factor, e is the electron charge, $m_{0}$ is the electron rest mass, and c is the velocity of light [119]. The slope of the $\mathrm{\Delta H}(f_{r})$ vs. $f_{r}$ relation allows for an estimation of the Gilbert damping constant α. Figure 14(a) depicts a representative FMR spectrum for a La${\;}_{1-x}$Sr${\;}_{x}$MnO${\;}_{3}$ thin film on PMN-PT(011) at various frequencies, from which the $\mathrm{\Delta H}(f_{r})$ vs. $f_{r}$ relation can be derived (Figure 14(b)). Das *et al*. examined the impact of electric poling on the Gilbert damping constant [119]. Prior to the application of an electric field (un-poled), the value of α is estimated to be 12$\times 10^{-3}$, while it decreases to 8.6$\times 10^{-3}$ for positive poling and 11$\times 10^{-3}$ for negative poling of the PMN-PT when a magnetic field sufficient to saturate the magnetization is applied along the magnetic easy axis. The asymmetric behavior with respect to the polarity of the electric field is likelyy due to a possible asymmetric piezostrain in PMN-PT(011) [26]. Since the damping constant is related to the magnetic anisotropy [120], the asymmetric electric field modulation of the damping constant results from a variation in the magnetic anisotropy induced by the asymmetric piezostrain. Nevertheless, both polarities of the electric field result in a decrease in the Gilbert damping constant. The results indicate that spin wave propagation and its velocity can also be controlled by an electric field in this manner.

### 4.6. Magnetic phase

**Figure 15.**
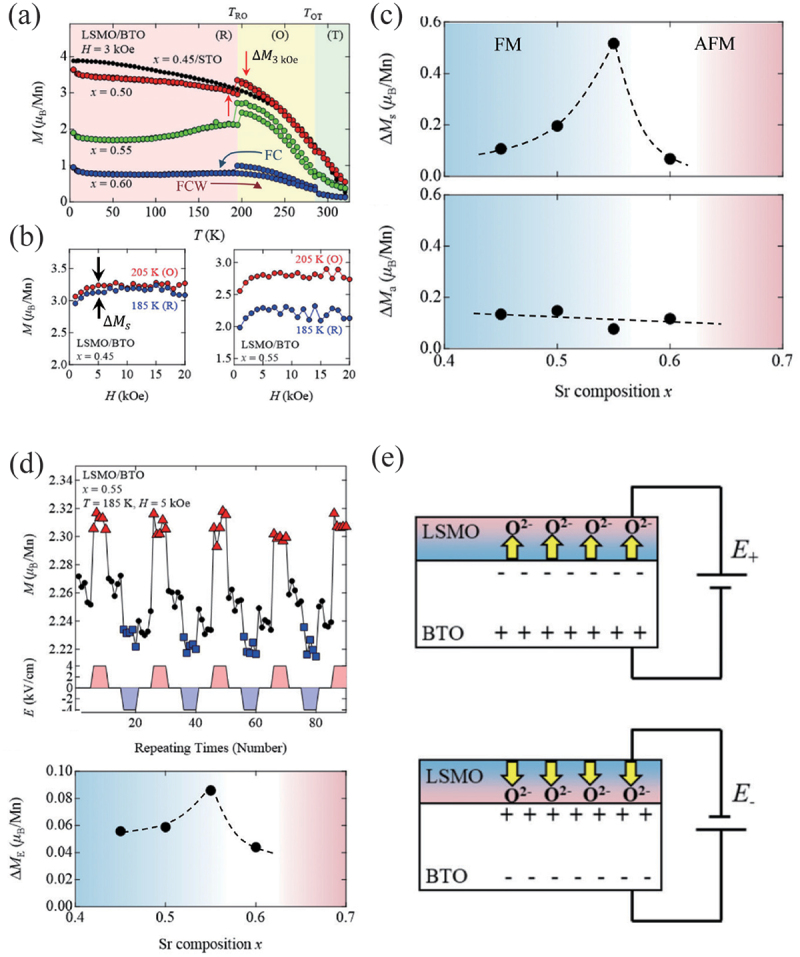
(a) Temperature dependence of magnetization of La${\;}_{1-x}$Sr${\;}_{x}$MnO${\;}_{3}$/BaTiO${\;}_{3}$ heterostructures. (b) Magnetization curves of La${\;}_{0.55}$Sr${\;}_{0.45}$MnO${\;}_{3}$/BaTiO${\;}_{3}$ and Magnetization curves of La${\;}_{0.45}$Sr${\;}_{0.55}$MnO${\;}_{3}$/BaTiO${\;}_{3}$. (c) Sr composition dependence of the difference in the saturation magnetization ($\Delta M_{s}$) and the magnetization due to magnetic anisotropy ($\Delta M_{s}$) between the orthorhombic phase and the rhombohedral phase of BaTiO${\;}_{3}$. (d) Magnetization switching of La${\;}_{0.45}$Sr${\;}_{0.55}$MnO${\;}_{3}$/BaTiO${\;}_{3}$ at T=185 K when an electric field is applied repeatedly, and Sr composition dependence of $\Delta M_{E}$. (e) Schematic illustration of oxygen diffusion in La${\;}_{1-x}$Sr${\;}_{x}$MnO${\;}_{3}$/BaTiO${\;}_{3}$ upon reversal of the electric field polarity. Reprinted with permission from [45]. Copyright 2023 AIP Publishing.

Manganese oxides La${\;}_{1-x}$Sr${\;}_{x}$MnO${\;}_{3}$ possess intriguing magnetic properties that exhibit a FM metal phase for 0.18<x<0.5 and an AFM metal phase for x>0.5 [121]. Given that the FM ordering arises from the double exchange interaction between Mn${\;}^{3+}$ and Mn${\;}^{4+}$ ions, the magnetic ordering is highly sensitive to lattice distortion and can be modulated by the inverse piezoelectric effect when La${\;}_{1-x}$Sr${\;}_{x}$MnO${\;}_{3}$ is grown on a FE substrate. Imura *et al*. investigated the impact of lattice distortion on the magnetic properties of La${\;}_{1-x}$Sr${\;}_{x}$MnO${\;}_{3}$/BaTiO${\;}_{3}$ heterostructures, employing techniques such as phase transition and inverse piezoelectricity of BaTiO${\;}_{3}$ [45]. BaTiO${\;}_{3}$ is a well-known FE material that exhibits successive structural phase transitions from the cubic phase to the tetragonal phase, the orthorhombic phase, and the rhombohedral phase at temperatures of 398 K, 278 K, and 183 K, respectively [6]. As illustrated in Figure 15(a), the magnetization displays sudden jumps at the structural phase transition temperatures. Given that the magnetization at 3 kOe is not saturated, the observed jumps in magnetization can be attributed to a change either in magnetic anisotropy or in saturation magnetization. In order to extract the variation of the saturation magnetization, the authors measured the difference in the saturation magnetization at 5 kOe just above (205 K) and below (185 K) the orthorhombic-rhombohedral phase transition, which they denote as $\Delta M_{s}$. $\Delta M_{s}$ and the corresponding magnetic anisotropy contribution, $\Delta M_{a}$, are plotted as a function of Sr composition in Figure 15(c), which demonstrates that the strain transfer-induced $\Delta M_{s}$ peaks at the phase boundary between the FM phase and the AFM phase. The saturation magnetization is also modulated by switching the polarity of an electric field ($\Delta M_{E}$), which the authors attribute to the oxygen migration as illustrated in Figure 15(e). When a positive electric field is applied, the induced negative polarization charges in the BaTiO${\;}_{3}$ at the interface repel the O${\;}^{2-}$ ions. Since a reduction in the amount of O${\;}^{2-}$ ions for positive electric fields corresponds to electron doping in the double exchange interaction, the application of the positive electric field stabilizes the FM correlation. On the other hand, when the polarity of the electric field is reversed, the positive polarization charges attract O${\;}^{2-}$ ions toward the interface, leading to the AFM correlation and the reduction in the saturation magnetization. Therefore, the electric field modulation of the saturation magnetization is likely due to partial conversion between the FM phase and AFM phase. In fact, the Sr composition dependence of the $\Delta M_{E}$ is the most significant near the FM-AFM phase boundary, similar to the strain transfer effect on $\Delta M_{s}$. A further example of electric field induced FM to AFM phase conversion has been reported in other systems such as FeRh/ferroelectric heterostructures. [21,122–125].

### 4.7. Superconductivity

**Figure 16.**
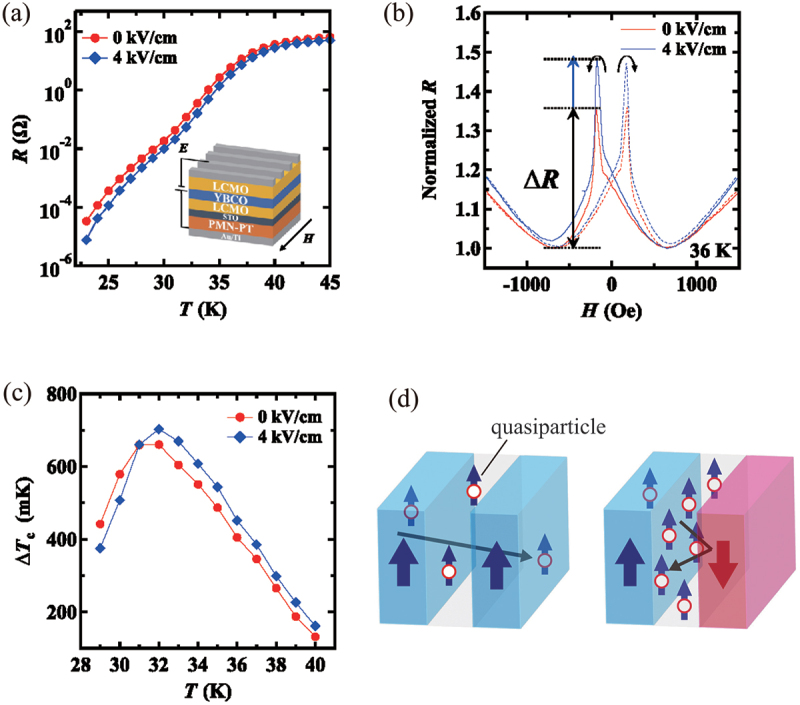
(a) Temperature dependence of resistance, (b) magnetic field dependence of normalized resistance, and (c) temperature dependence of $\Delta T_{C}$ for a spin valve structure La${\;}_{0.67}$Ca${\;}_{0.33}$MnO${\;}_{3}$/YBa${\;}_{2}$Cu${\;}_{3}$O${\;}_{7}$/La${\;}_{0.67}$Ca${\;}_{0.33}$MnO${\;}_{3}$ at electric fields of 0 and 4 kV/cm. (d) Schematic diagram of the quasiparticles accumulation in YBa${\;}_{2}$Cu${\;}_{3}$O${\;}_{7}$ for the antiparallel orientation. Reprinted with permission from [74]. Copyright 2024 the Authors. APL Mater. published by AIP Publishing.

As the last example of applications of artificial multiferroic heterostructures, we demonstrate electric field effect on superconductivity. As previously stated, the magnetization orientation of a FM film on a FE substrate can be controlled by an electric field. This enables the manipulation of the superconducting phase transition temperature, $T_{C}$, in superconducting spin valve structures. In a very recent study, Kikuta *et al*. demonstrated the ability to control the superconducting transition temperature of YBa${\;}_{2}$Cu${\;}_{3}$O${\;}_{7}$ in the spin valve structures La${\;}_{0.67}$Ca${\;}_{0.33}$MnO${\;}_{3}$/YBa${\;}_{2}$Cu${\;}_{3}$O${\;}_{7}$/La${\;}_{0.67}$Ca${\;}_{0.33}$MnO${\;}_{3}$ by an electric field [74]. As illustrated in Figure 16(a), the temperature dependence of the resistance shifts towards higher temperatures upon the application of an electric field, indicating an enhancement in the superconducting transition temperature. Additionally, the magnetic field dependence of the resistance in the normal state in the immediate vicinity of the superconducting transition temperature exhibits pronounced peaks at ±200 Oe, with the peaks becoming more pronounced by 33% in the presence of an electric field as shown in Figure 16(b). The observed peaks correspond to a decrease in the $T_{C}$, indicating that the $T_{C}$ decreases when the two FM La${\;}_{0.67}$Ca${\;}_{0.33}$MnO${\;}_{3}$ are oriented antiparallel to each other. The authors attribute this to possible spin-dependent scattering effects of the quasiparticles, as previously reported [126,127]. Since the two La${\;}_{0.67}$Ca${\;}_{0.33}$MnO${\;}_{3}$ are antiparallel, spin polarized quasiparticles traversing the interface are blocked by the antiparallel La${\;}_{0.67}$Ca${\;}_{0.33}$MnO${\;}_{3}$ as shown in Figure 16(d), leading to an increase in the density of quasiparticles and a consequent decrease in $T_{C}$. They also estimated the change in $T_{C}$ for the antiparallel magnetization orientation, $\Delta T_{C}$, from the change in the resistance, ΔR. The temperature dependence of $\Delta T_{C}$ shown in Figure 16(c) is due to the balance between the densities of quasiparticles and Cooper pairs in YBa${\;}_{2}$Cu${\;}_{3}$O${\;}_{7}$. More interestingly, the application of an electric field is shown to increase the $\Delta T_{C}$ by 6%, presumably due to an increase in the magnetization of La${\;}_{0.67}$Ca${\;}_{0.33}$MnO${\;}_{3}$ when an electric field is applied. These combined results suggest that the electric field effect on $T_{C}$ may offer a potential avenue for energy-efficient control of the Curie temperature of a superconductor.

## 5. Summary and future perspectives

We have reviewed recent research progress in artificial multiferroic heterostructures, with a focus on the energy-efficient control of magnetic and transport properties by an electric field. In order to integrate a multiferroic heterostructure into real memory applications, it is necessary to reduce the voltage, which represents a significant challenge for the data writing process in multiferroic heterostructures. In accordance with the empirical Janovec-Kay-Dunn law, the FE coercivity, $E_{c}$, scales with the film thickness, d, as $\propto d^{-2/3}$ [128]. Therefore, a significantly thinner and higher-quality FE layer is necessary. One potential solution is the use of HfO${\;}_{2}$-based FE thin films, which have already been integrated as gate insulators in silicon technology. In this regard, Dmitriyeva *et al*. have demonstrated a substantial reduction in the switching voltage, down to 2.5 V, in Co/Ni/Hf${\;}_{0.5}$Zr${\;}_{0.5}$O${\;}_{2}$ [129]. A first-principles calculation of ME-induced magnetic moment has been made for a Ni/HfO${\;}_{2}$/Ni structure, with a value of 1.4 $\mu_{B}$, through the reversal of FE polarization direction [130]. Another architectural design developed by Intel, which incorporates both the inverse Rashba Edelstein effect and the ME effect, has demonstrated significant potential for ultra-low power switching operations using BiFeO${\;}_{3}$-based multiferroic heterostructures, achieving a switching energy density of 1–10 μJ/cm${\;}^{2}$, comparable to aJ-class nonvolatile memories archtecture [131–134]. Concurrently, the switching voltage is markedly diminished to a range of 200 mV, which is highly encouraging for the practical deployment of these devices beyond the domain of fundamental research. A recent report by Jiang *et al*. demonstrates the ultra-low voltage switching of FE polarization in BaTiO${\;}_{3}$ at a voltage of less than 100 mV, representing a remarkable advancement in the fabrication of high-quality ferroelectric thin films for use in artificial multiferroic heterostructures. Two-dimensional multiferroics could also provide an exciting route to creating new types of multiferroic heterostructures that enable electric field control of magnetism at extremely low voltages [135,136].

As another platform for non-volatile information carriers in spintronic devices, topological spin textures, *e.g*. magnetic skyrmion and bimeron have also attracted much attention for the development of energy-efficient memory device applications. Wang *et al*. have demonstrated electric field control of magnetic skyrmions in [Pt/Co/Ta]_*n*_/PMN-PT artificial multiferroic heterostructures, achieving stripe-skyrmion-vortex multistate switching [38]. Electric field manipulation of magnetic skyrmions and bimerons has also been reported in MnBi${\;}_{2}$Se${\;}_{2}$Te${\;}_{2}$/FE In${\;}_{2}$Se${\;}_{3}$ multiferroic heterostructures by reversing the FE polarization using first-principles calculations [137]. Since topologically protected objects are small and stable, these topological multiferroic heterostructures hold great promise for integration into low-power, high-density spintronic devices.

As we have seen, electric field control of magnetization orientation surpasses the energy efficiency of the current-based control method based on spin transfer torque and spin orbit torque. However, if a spin-triplet superconducting current carrying spin angular momentum could be used in the current-based approach, the problem of Joule heating can be circumvented. Since the conversion of a spin-singlet super current into a spin-triplet super current has been demonstrated using an inhomogeneous or non-collinear spin configuration at the interfaces of spin-singlet superconductor/FM materials [138–141], the electric field manipulation of the inhomogeneous spin configuration could lead to a novel approach to generate a spin-triplet super current by integrating artificial multiferroic heterostructures, although this approach has not yet been achieved.

In addition to memory applications, numerous other potential applications of artificial multiferroic heterostructures have been proposed, including energy harvesting [142] and magnetic field sensors [143]. When the FM material forming the artificial multiferroic heterostructure is deformed by the magnetostriction effect caused by microwaves, it is converted into a voltage in the piezoelectric layer and subsequently recovered as electricity. A comparable mechanism may be employed as a magnetic field sensor. Furthermore, the control of artificial multiferroic heterostructures by light has recently been reported [144]. It has been demonstrated that the FE domain structure of BaTiO${\;}_{3}$ can be controlled by irradiation with light, and that the magnetism of Ni/BaTiO${\;}_{3}$ can be modulated by the magnetoelastic effect. This clearly indicates that by modulating one of the ferromagnetism, ferroelectricity, and ferroelasticity of the multiferroic heterostructures with light, it is possible to control the combined other properties simultaneously. It is expected that this will be applied to a wide range of electronics. Based on these observations, we postulate that artificial multiferroic heterostructures will offer a plethora of prospects for future low-power device implementations. Nevertheless, certain facets of polarization fatigue in ferroelectrics [145] and retention failure [146] in non-volatile multiferroic ME memory devices remain formidable challenges.
